# Psychoeducation for Youth at Clinical High‐Risk of Psychosis

**DOI:** 10.1111/eip.70274

**Published:** 2026-10-02

**Authors:** Jean Addington, Lu Liu, Cari Jahraus, Erin Jones, Joyce Singh

**Affiliations:** ^1^ Department of Psychiatry, Hotchkiss Brain Institute University of Calgary Calgary Alberta Canada

**Keywords:** clinical high‐risk, clinical trial, psychoeducation, psychosis

## Abstract

**Introduction:**

Psychoeducation may be a useful first‐step intervention for youth at clinical high risk of developing psychosis (CHR). The aim of this study was to describe clinical improvement after receiving psychoeducation and adherence to the treatment.

**Methods:**

The study included 54 participants who began the treatment and 48 completed a follow‐up assessment 2–6 months after baseline. Clinical assessments included measures of attenuated psychotic symptoms (APS), negative symptoms, depression, anxiety, distress and social and role functioning. Treatment consisted of eight individual sessions of psychoeducation.

**Results:**

At the follow‐up assessments, significant improvements were observed in total ratings on APS (*p* < 0.0001, effect size (ES) = 0.36), depression and anxiety ratings on the CDSS (*p* < 0.001, ES = 0.51) and the OASIS (*p* < 0.05, ES = 0.31), the K‐10 distress scale (*p* < 0.01, ES = 0.36) and defeatist beliefs (*p* < 0.01, ES = 0.34). For negative symptoms, significant improvements were observed in blunted affect (*p* < 0.01, ES = 0.45) and avolition (*p* < 0.05, ES = 0.21). No improvements were observed in social or role functioning. Fifty‐nine percent completed all psychoeducation sessions, and 18.5% chose to attend fewer sessions due to existing knowledge. Only 7.4% dropped out, while the remaining 14.7% left early but provided explanations for doing so.

**Conclusions:**

Generally, participants attended most of the sessions, with limited dropout. Clinical improvement was observed but cannot necessarily be attributed to the psychoeducation since this was an open trial. However, this appears to be a valuable and easy‐to‐deliver intervention that can be offered in a real‐world clinical setting.

**Trials Registration:** Clinical Trials.gov identifier NCT04829292.

## 1 Introduction

For the past 25 years, a major focus of schizophrenia research has been youth at clinical high risk (CHR) for psychosis, with the primary goal of preventing the transition to a full‐blown psychotic disorder. Although CHR individuals may experience changes in their behaviour and thinking, their symptoms, including unusual thoughts, perceptual abnormalities and suspicious ideas, are attenuated and do not reach the threshold for psychosis. Approximately 20% are at increased risk of developing a psychotic disorder. However, even among those who do not transition to psychosis, persistent attenuated psychotic symptoms (APS), mood and anxiety disorders and impairments in social and role functioning remain common (Addington et al. 2019). Furthermore, CHR individuals vary widely in symptom severity, comorbidities and functional impairment (Addington et al. 2025). Thus, the heterogeneity of CHR youth poses significant challenges for treatment planning. These findings underscore the need to develop and evaluate accessible interventions that address the ongoing symptoms and functional challenges faced by CHR youth. Unfortunately, empirical data regarding psychosocial treatments for CHR youth remain limited, with only a small number of randomized clinical trials (RCTs) conducted to date (Mei et al. 2021; Minichino et al. 2025).

It has been suggested that stepped care may be a useful approach to address the clinical heterogeneity observed in CHR youth. The first reported project for CHR was the Staged Treatment in Early Psychosis Trial (McGorry et al. 2023), a sequential multiple assignment randomized trial (SMART) in which the treatment modality and/or the amount of treatment is personalized, based on the individual's clinical presentation or treatment response. Two small open trials followed (Hamilton et al. 2024; Shapiro et al. 2026), as well as a report using secondary analyses (Cosgrove et al. 2026). The common element across all these stepped‐care programs was the use of psychoeducation as the first step. Overall, there is some preliminary evidence that these programs may contribute to improvements in various client outcomes, including reduced psychological distress.

In a recent scoping review of 33 studies, Herrera and colleagues concluded that psychoeducation is recommended in the treatment of CHR youth and is a preferred treatment option among this group (Herrera et al. 2023). Many CHR programs report offering psychoeducation; however, detailed information about the content, methods and delivery of psychoeducation is often lacking in reports describing its provision in real‐world settings. Herrera et al. (2025) conducted a feasibility and pilot study of a Brief Educational Guide for Individuals in Need (BEGIN) for CHR individuals. Results indicated improvements in CHR knowledge, and symptom monitoring skills, and increased therapy motivation. Overall, the trial was feasible and acceptable.

Thus, as a low‐risk and scalable intervention, psychoeducation offers a practical and developmentally appropriate first step in treatment, providing information about symptoms, risk status, coping strategies and additional treatment options (Herrera et al. 2025). Psychoeducation, when offered as an early intervention for CHR youth, has been shown to increase an individual's sense of agency by helping them feel more in control of their mental health and enhancing their ability to be more proactive in making choices and planning next steps (Herrera et al. 2025).

Collectively, the literature supports psychoeducation as a promising early intervention while underscoring the need for more rigorous evaluation. Empirical data evaluating psychoeducation as a discrete, manualized intervention in CHR samples remain limited, particularly with respect to clinical outcomes, engagement and adherence. Given that early intervention is crucial in shaping illness trajectories, understanding how CHR youth engage with, select and adhere to treatments is essential for advancing care.

This paper reports on the first step of an open trial offering a staged approach to treatment that examines clinical outcomes and treatment choices for CHR youth. The design and methods of the broader project have been detailed in a previous publication (Addington et al. 2025). This first step consists of an eight‐session psychoeducation program. The aim of this paper is to describe clinical improvement following psychoeducation and treatment adherence.

## 2 Methods

### 2.1 Design

In this 5‐year open trial conducted at the University of Calgary, Canada, participants were recruited over a 3‐year period. Assessments occurred at baseline and at 2‐, 6‐, 12‐ and 18‐months.

### 2.2 Participants

Recruitment procedures included the following steps: (1) dissemination of informational materials outlining the project, (2) presentations to mental health agencies that serve youth at risk of psychosis, (3) engagement with school and post‐secondary counselling services and (4) advertising through local newspapers and community magazines.

Participants were between the ages of 12 and 29. After an initial phone screen, potentially eligible participants attended a face‐to‐face assessment to determine whether they met study inclusion criteria. Eligible and interested participants provided written informed consent. The study was approved by the University of Calgary Conjoint Health Research Ethics Board. Participants provided informed consent or assent, with parental consent obtained for minors.

Exclusion criteria included (i) meeting criteria for a current or lifetime psychotic disorder, including affective psychoses, (ii) an IQ < 70, (iii) a history of a central nervous system disorder, or (iv) psychosis‐risk symptoms that were clearly caused by another disorder. Other non‐psychotic DSM‐5 disorders were not exclusionary, provided the disorder did not account for the psychosis‐risk symptoms. Using the Structured Interview for Psychosis‐risk Syndromes (SIPS) (McGlashan et al. 2010), participants either currently met or had met within the past 2 years the Criteria of Psychosis‐Risk Syndromes (COPS). Participants who did not presently meet COPS were required to exhibit current APS, as assessed by the Scale of Psychosis‐risk Symptoms (SOPS), thereby meeting criteria for persistent APS.

### 2.3 Measures

Participant demographic information included age, sex and race/ethnicity.

The SIPS (McGlashan et al. 2010) was used to ascertain psychosis‐risk criteria and to assess APS. The APS subscale comprises five symptoms (unusual thought content, suspiciousness, grandiosity, perceptual abnormalities and disorganized communication). The Structured Clinical Interview for DSM‐5 (SCID) was used to determine comorbid diagnoses (First et al. 2015).

Negative symptoms (anhedonia, avolition, asociality, alogia, blunted affect) were assessed with the Negative Symptom Inventory‐Psychosis Risk (NSI‐PR) (Strauss et al. 2025); depression was assessed with the Calgary Depression Scale for Schizophrenia (CDSS) (Addington et al. 1993, 2014) and anxiety with the Overall Anxiety Severity and Impairment Scale (OASIS) (Norman et al. 2006).

The Global Functioning: Social (GF:S) and the Global Functioning: Role (GF:R) scales were used to measure functioning (Auther et al. 2006; Cornblatt et al. 2007). Additional measures included the Defeatist Performance Attitude Scale (DPAS), a 15‐item self‐report scale assessing defeatist attitudes on a 1–7 Likert scale, with higher scores indicating more severe defeatist beliefs (Cane et al. 1986) and the Kessler Distress Scale (K10) (Kessler et al. 2002), a 10‐item scale that yields a global measure of distress.

The Wechsler Abbreviated Scale of Intelligence (WASI‐2) vocabulary and matrix reasoning subtests (Wechsler 2011) were used to assess participants' intellectual functioning.

The schedule of measures for the first 6 months is presented in Table 1.

**TABLE 1:** Schedule of clinical measures.

Measure	Baseline	2‐months	6‐months
Demographics	√		
Structure clinical interview for DSM‐5	√		
Scale of psychosis‐risk symptoms	√	√	√
Calgary depression scale	√	√	√
Negative symptom inventory‐psychosis risk	√	√	√
Global functioning scales: social and role	√	√	√
Overall anxiety severity and impairment scale	√	√	√
K10‐distress scale	√	√	√
Defeatist performance beliefs	√	√	√
Wechsler abbreviated scale of intelligence	√		

### 2.4 Treatment

Treatment reported in this paper consists of eight sessions of individual psychoeducation. In delivering the psychoeducation, adaptations were made to ensure developmental appropriateness across the participant age range. Although the psychoeducation material was standardized and covered the same core topics, therapists tailored examples, discussions and learning activities to participants' life circumstances and presenting concerns. For example, the communication skills section might focus on parent–child relationships for younger participants, workplace or romantic relationships for older participants, or other contexts most relevant to the individual.

The psychoeducation sessions addressed a broad range of topics, with the flexibility to reduce or omit a session if the participant and therapist jointly determined that the participant already had sufficient knowledge in a specific area or if the topic was not relevant for the participant. For instance, a participant exhibiting good sleep habits might not require a dedicated session on sleep hygiene. Alternatively, reducing time on one topic could allow more time to focus on areas of greater concern. Regardless, all material remained available to participants in the handouts provided.

Initial training sessions were conducted with the therapists to ensure they were familiar with all the material in the manual. Biweekly supervision sessions with J. Addington were held with the study therapists, who were master's‐level clinicians experienced in working clinically with CHR participants. An outline of all psychoeducation sessions is presented in the Supplementary Material. The manual is available on request.

### 2.5 Procedures

Since this is an ongoing trial, this paper includes assessments conducted at baseline, and at 2‐, and 6‐months post baseline. Clinical raters were experienced, trained on all clinical measures and participated in regular reliability checks.

If a participant transitioned to psychosis during the study, appropriate psychiatric care was provided.

### 2.6 Statistical Analysis

Descriptive analyses were conducted for continuous and categorical variables using means, standard deviations and frequencies (see Tables 2 and 3).

**TABLE 2:** Demographics.

Variable	CHR (*n* = 54), Mean (SD) *N* (%)
Age	18.89 (4.42)
Years of education	11.33 (2.66)
Sex	
Male	22 (40.74%)
Female	32 (59.26%)
Race^a^	
Indigenous	7 (12.96%)
Southeast Asian	1 (1.85%)
South Asian	4 (7.41%)
Black	3 (5.56%)
White	33 (61.11%)
Interracial	6 (11.11%)
Living arrangement	
With family	46 (85.19%)
With spouse/partner	4 (7.41%)
Living alone	3 (5.56%)
Group/boarding home	1 (1.85%)
Highest education level	
Some grade school	29 (53.7%)
High school	21 (38.89%)
College	1 (1.85%)
Technical school	1 (1.85%)
University	2 (3.7%)
First degree relative with a psychotic disorder	4 (7.41%)

^a^ Southeast Asian Includes: East Indian, Pakistani, Sri Lankan; South Asian Includes: Cambodian, Indonesian, Vietnamese.

**TABLE 3:** Diagnoses.

	CHR (*n* = 54), *N* (%)
SCID diagnosis	
Depression	32 (59.3%)
Any anxiety disorder	29 (53.7%)
Obsessive compulsive disorder	6 (10.4%)
Alcohol use disorder	5 (10.4%)
Cannabis use disorder	5 (10.4%)
SIPS syndromes	
APPS current progression	24 (44.4%)
APPS current persistence	29 (53.7%)
GRD current progression	1 (1.9%)

Abbreviations: APPS, attenuated positive psychotic symptoms; GRD, genetic risk and deterioration; SCID, structured clinical interview for DSM‐5; SIPS, structured interview for psychosis‐risk syndromes.

Changes from baseline to 2 months were examined using generalized linear models in 48 participants. For 11 participants who either missed their 2‐month assessment or did not complete their psychoeducation sessions until several months later, their 6‐month assessment was used in this analysis.

## 3 Results

Fifty‐four participants were recruited for this project. Six participants did not complete the 2‐ and 6‐month follow‐up assessments. Of these, one participant completed all 8 psychoeducation sessions but could not be reached for any follow‐ups. For the other four participants, two dropped out after two treatment sessions and could not be reached, one began residential treatment after two sessions and left the study and one discontinued after three sessions to seek treatment elsewhere. The sixth participant completed a course of CBT as well as the psychoeducation by the first follow‐up, and thus any improvement could not be attributed to psychoeducation alone. Therefore, only 48 participants were included in any data analyses examining follow‐up data. See Figure 1 for Consort Diagram.

**FIGURE 1:**
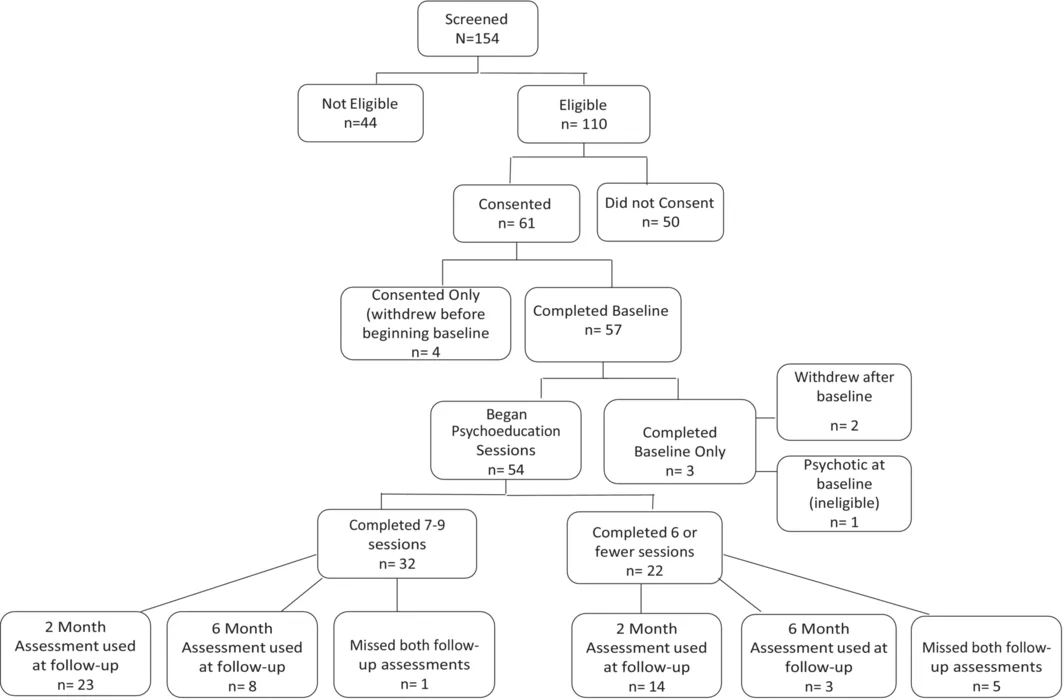
Consort diagram.

Demographics of the sample are presented in Table 2. DSM‐5 diagnoses and SIPS criteria are presented in Table 3.

Thirty‐seven participants completed the 2‐month assessment. Eleven participants either missed the 2‐month assessment or had not completed their psychoeducation sessions in time; thus the 6‐month follow‐up was used instead.

Psychoeducation consisted of eight sessions. Of the 54 participants who started the trial, 32 (59%) completed 7–9 sessions, while 22 completed six or fewer sessions. Among these 22 participants, 10 completed 3–6 sessions because they reported already having sufficient knowledge; 2 completed 3–4 sessions due to work and school commitments; 6 attended only a few sessions to focus on therapy received elsewhere; and 4 completed 2–4 sessions but could not be recontacted.

Change over time was examined using generalized linear models in 48 participants. Total SOPS score significantly improved from baseline to follow‐up (*p* < 0.0001, effect size = 0.36), primarily driven by reductions in unusual thought content, suspiciousness and perceptual abnormalities. Significant improvements were also observed over time in depression and anxiety ratings on the CDSS (*p* < 0.001, effect size [ES] = 0.51) and the OASIS (*p* < 0.05, ES = 0.31), the K‐10 distress scale (*p* < 0.01, ES = 0.36) and defeatist beliefs (*p* < 0.01, ES = 0.34). For the five negative symptom domains, small but significant improvements were observed in blunted affect (*p* < 0.01, ES = 0.45) and avolition (*p* < 0.05, ES = 0.21). No improvements were observed in social or role functioning. These findings are presented in Table 4.

**TABLE 4:** Generalized linear model for changes over time.

Variables	Baseline (*n* = 48) Mean (SE)	Follow‐up visit (*n* = 48) Mean (SE)	Effect size Cohen's *d*
Scale of psychosis risk symptoms			
Unusual thought content	3.27 (0.199)	2.94 (0.202)*	0.24
Suspiciousness/persecutory ideas	2.94 (0.198)	2.52 (0.201)**	0.30
Grandiose ideas	0.38 (0.125)	0.35 (0.113)	0.04
Perceptual abnormalities	3.08 (0.178)	2.71 (0.195)*	0.28
Disorganized communication	1.96 (0.165)	1.67 (0.164)**	0.25
Total score	11.63 (0.538)	10.19 (0.597)****	0.36
Negative symptom inventory‐psychosis risk			
Avolition	2.35 (0.165)	2.10 (0.165)*	0.21
Asociality	2.28 (0.154)	2.33 (0.134)	−0.05
Anhedonia	2.11 (0.198)	1.92 (0.183)	0.14
Blunted Affect	0.60 (0.131)	0.27 (0.072)**	0.45
Alogia	0.52 (0.139)	0.34 (0.114)	0.21
CDSS	8.21 (0.767)	5.63 (0.694)***	0.51
OASIS	9.84 (0.654)	8.45 (0.651)*	0.31
Defeatist Performance Beliefs	58.18 (2.633)	52.38 (2.231)**	0.34
K10‐Distress Scale	32.73 (1.397)	29.29 (1.389)**	0.36
Global Functioning: Social Scale	6.58 (0.192)	6.60 (0.210)	−0.01
Global Functioning: Role Scale	5.71 (0.381)	5.54 (0.362)	0.07

*Note:* Mean represents the least squares means estimated by the Generalized linear models, SE represents the standard error of the mean.

Abbreviations: CDSS, Calgary Depression Scale for Schizophrenia; OASIS, Overall Anxiety Severity and Impairment Scale.

* *p* < 0.05.

** *p* < 0.01.

*** *p* < 0.001.

**** *p* < 0.0001.

We also examined whether the number of sessions attended impacted change over time by repeating the primary generalized linear models with session count included as a covariate. These results were consistent with those presented in Table 4. See Supplementary Material Table 1.

Next, we examined whether the number of sessions attended was associated with any clinical variables. The number of sessions was moderately correlated with WASI IQ, indicating that participants with higher IQ tended to complete fewer sessions, and with SOPS total score, indicating that higher symptom severity was associated with attending more sessions. No other demographic, clinical, or symptom measures were significantly associated with the number of sessions (Table 5).

**TABLE 5:** Pearson correlations between number of sessions attended and demographic and clinical variables (*n* = 54).

Variables	*r*	*p*
Age	0.00	0.99
Years of education	−0.01	0.93
Scale of Psychosis Risk Symptoms Total Score	0.29^a^	0.03
CDSS	−0.03	0.82
OASIS	0.14	0.34
K10‐distress scale	0.00	0.99
IQ	−0.38^a^	0.01

*Note:* Pearson's correlation coefficients (*r*) are shown. *p* values are two‐tailed.

Abbreviations: CDSS, Calgary Depression Scale for Schizophrenia; IQ, Intelligence Quotient; OASIS, Overall Anxiety Severity and Impairment Scale.

^a^ *p* < 0.05.

## 4 Discussion

This paper describes the outcome and adherence of a sample of CHR youth following an 8‐session individual psychoeducation package. We first examined change over time on the clinical measures. For the 48 participants with follow‐up, significant improvements in APS, depression, anxiety and distress were observed. There was some improvement in avolition and blunted affect, which may have been related to the improvement in depression. However, post hoc longitudinal change‐score correlations (follow‐up minus baseline) confirmed a significant trend linking reductions in depression with reductions in blunted affect, although this synchronous improvement was not statistically significant for avolition.

There was no improvement in either social or role functioning. This was not surprising, as improvements in social and role functioning are not always seen in CHR youth (Addington et al. 2019), and 2 months may be too short a time frame for such change to emerge.

Secondly, we aimed to better understand treatment adherence. Fifty‐nine percent completed all sessions, with only less than 8% dropping out without any reason. Thus, relative to other studies, the dropout rate was relatively low (McGorry et al. 2023).

Thirdly, only the SOPS total and IQ were related to the number of sessions attended. Participants with more severe APS symptoms attended more sessions, possibly because they felt a greater need for support or required more time in treatment. In contrast, those with less severe symptoms may have progressed more quickly or been less motivated to continue. The IQ findings are more difficult to interpret, given the small sample. It is possible that participants with higher IQ were able to engage with and assimilate the material more quickly, or that higher IQ was associated with lower symptom severity overall. Supporting this, cross‐sectional analyses showed that higher baseline IQ was associated with lower symptom severity as per the SOPS total score. However, IQ did not significantly correlate with the magnitude of longitudinal symptom change, suggesting that higher IQ was associated with lower baseline symptom severity rather than greater symptom improvement over time.

Much of the current research examining psychological treatments for CHR youth occurs within well‐established research centres or clinical programs that are specifically designed for those at risk. In smaller centres, individuals at risk of psychosis may be involved in youth mental health programs or general community mental health centre settings where only small numbers of CHR youth are identified. This psychoeducation package is readily available, easy to administer and may be a valuable option for CHR youth in settings without specialized programs.

### 4.1 Limitations

There are several limitations to this project. First, this was an open trial, and it is not possible to determine whether clinical improvements were directly attributable to psychoeducation. We had five participants who missed the 2‐month assessment and six who had completed less than half of their sessions by that time point. For these participants, the 6‐month assessment was used to examine change over time. However, none of these participants received any additional treatment between the scheduled 2‐month assessment and their 6‐month follow‐up.

### 4.2 Recommendations

In delivering a psychoeducation package, it may be important to consider which sessions are most relevant for different CHR clients. For clients already engaged with other therapists, coordination regarding which provider delivers specific content may be helpful. Finally, although we were not successful in retaining the two participants who reported work and school conflicts, maintaining flexibility in scheduling sessions should always be considered.

## Funding

This study was funded by a grant from the Alberta Children's Hospital Foundation to J Addington.

## Conflicts of Interest

The authors declare no conflicts of interest.

## Supporting information


**Data S1:** Supporting Information.

**Supplementary Table 1.** Generalized linear models for change over time controlling for number of sessions.

## Data Availability

Data will be available once the study is completed.
