# Supplementary material for: Psychoeducation for Youth at Clinical High‐Risk of Psychosis
Source: Early Interv Psychiatry. 2026 Oct 2;20(10):e70274. doi: 10.1111/eip.70274 (PMC13633424; doi:10.1111/eip.70274)
Supplement: Supplementary file 1 [file EIP-20-0-s001.docx]

**Supplementary Material**

**Psychoeducation**

The purpose of this step is to provide the participant or client with support and information about mental health in general, with a specific focus on psychosis risk, and including strategies to help cope with stress and adversity. Topics covered in the 8 sessions include:

- Psychosis risk symptoms and risk categories
- Stress, the role it plays in mental illness and ways to help manage it
- Sleep, its importance and how to establish good habits
- Other factors that can impact mental health including substance use and comorbidities
- Coping skills and strategies
- Identifying and leveraging protective factors and individual strengths
- Communication skills and self-advocacy

As you explore these topics, allow the participant or client to reflect on their symptoms and experiences. Your role is to provide an open and supportive environment in which to answer their questions and offer useful evidence-based information.

PowerPoint slides are available to provide sessions over zoom. These slides can also be displayed using an iPad or laptop for in-person sessions, or they can be printed off. Notes have been provided with each slide to help with session preparation, and to guide sessions. Additional handouts are also available to share with participants as needed.

Session 1 – Introductions and What to expect

Session 2 – Psychosis and Psychosis Risk

Session 3 – Managing Stress

Session 4 – Other Factors that Impact our Mental Health

Session 5 – Coping Skills

Session 6 – Protective Factors and Individual Strengths

Session 7- Communication Skills and Self Advocacy

Session 8- Review, Final Questions, Resources and Feedback
