# Supplementary material for: Psychoeducation for Youth at Clinical High‐Risk of Psychosis
Source: Early Interv Psychiatry. 2026 Oct 2;20(10):e70274. doi: 10.1111/eip.70274 (PMC13633424; doi:10.1111/eip.70274)
Supplement: Supplementary file 2 [file EIP-20-0-s002.docx]

**Supplementary Table 1: Generalized linear models for change over time controlling for number of sessions.**

| Variables | | Baseline (n=48)  *Mean (SE)* | 2-Month Visit (n=48)  *Mean (SE)* | Effect Size  Cohen's d |
| --- | --- | --- | --- | --- |
| Scale of Psychosis Risk Symptoms | |  |  |  |
|  | *Unusual thought content* | 3.27 (0.196) | 2.94 (0.197)* | 0.25 |
|  | *Suspiciousness/persecutory ideas* | 2.94 (0.198) | 2.52 (0.202)** | 0.30 |
|  | *Grandiose ideas* | 0.38 (0.123) | 0.35 (0.110) | 0.04 |
|  | *Perceptual abnormalities* | 3.08 (0.177) | 2.71 (0.198)* | 0.28 |
|  | *Disorganized communication* | 1.96 (0.166) | 1.67 (0.165)** | 0.25 |
|  | *Total score* | 11.63 (0.518) | 10.19 (0.592)**** | 0.37 |
|  | |  |  |  |
| Negative Symptom Inventory-Psychosis Risk | |  |  |  |
|  | *Avolition* | 2.35 (0.167) | 2.10 (0.167)* | 0.20 |
|  | *Asociality* | 2.28 (0.154) | 2.33 (0.135) | -0.05 |
|  | *Anhedonia* | 2.11 (0.201) | 1.92 (0.184) | 0.14 |
|  | *Blunted Affect* | 0.60 (0.133) | 0.27 (0.072)** | 0.45 |
|  | *Alogia* | 0.52 (0.142) | 0.34 (0.113) | 0.21 |
|  | |  |  |  |
| CDSS | | 8.21 (0.776) | 5.63 (0.698)*** | 0.50 |
| OASIS | | 9.84 (0.647) | 8.48 (0.658)* | 0.30 |
| Defeatist Performance Beliefs | | 58.16 (2.663) | 52.34 (2.255)** | 0.34 |
| K10-Distress Scale | | 32.73 (1.406) | 29.42 (1.369)* | 0.34 |
| Global Functioning: Social Scale | | 6.58 (0.186) | 6.60 (0.214) | -0.01 |
| Global Functioning: Role Scale | | 5.71 (0.385) | 5.54 (0.366) | 0.07 |

*Abbreviations: Mean represents the least squares means estimated by the Generalized linear models, SE represents the standard error of the mean. CDSS, Calgary Depression Scale for Schizophrenia; OASIS, Overall Anxiety Severity and Impairment Scale.*

*Significance: *p < 0.05, **p < 0.01, ***p < 0.001, ****p<0.0001*
